# Compressive Sensing Image Sensors-Hardware Implementation

**DOI:** 10.3390/s130404961

**Published:** 2013-04-12

**Authors:** Mohammadreza Dadkhah, M. Jamal Deen, Shahram Shirani

**Affiliations:** Department of Electrical and Computer Engineering, McMaster University, Hamilton, ON L8S 4K1, Canada; E-Mails: dadkham@mcmaster.ca (M.D.); shirani@mcmaster.ca (S.S.)

**Keywords:** compressive sensing, image sensor, CS-CMOS imager, coded exposure, multiple-camera, high frame rate, video coding

## Abstract

The compressive sensing (CS) paradigm uses simultaneous sensing and compression to provide an efficient image acquisition technique. The main advantages of the CS method include high resolution imaging using low resolution sensor arrays and faster image acquisition. Since the imaging philosophy in CS imagers is different from conventional imaging systems, new physical structures have been developed for cameras that use the CS technique. In this paper, a review of different hardware implementations of CS encoding in optical and electrical domains is presented. Considering the recent advances in CMOS (complementary metal–oxide–semiconductor) technologies and the feasibility of performing on-chip signal processing, important practical issues in the implementation of CS in CMOS sensors are emphasized. In addition, the CS coding for video capture is discussed.

## 1. Introduction

Image sensors are of increasing importance in applications such as biomedical imaging, sensor networks, hand-held digital cameras, as well as cameras in cell phones, computers, and for ***c***losed ***c***ircuit television (CCTV) monitoring systems. The increasing demand for both high resolution and high frame rate cameras leads to a large amount of image data. With these large amounts of data, fast and accurate image compression algorithms to represent the data in a compact form are required. Typically, compression algorithms use the spatial and temporal correlations in image and video signals to remove redundant information while concurrently keeping the essential features intact. Different algorithms such as Huffman, arithmetic, dictionary, differential, sub-band and wavelet-based coding as well as quantization methods have been presented [1]. These algorithms perform the compression after the image acquisition.

In the compressive sensing (CS) method [2–7], instead of sensing the entire image and then subsequently removing redundant information during the compression step, only the required or non-redundant information is sensed. The CS paradigm has attracted increased interest in past decade because it intrinsically avoids sensing redundant information that exists in image or video data. In CS, a number of random projections of the image are being sensed as the compressed version of the image, thus leading to the faster image acquisition system.

The CS method has also found extensive applications because of its impact in decreasing the number of the sensors for a given image resolution and reducing the image acquisition time. Some of the main applications of the CS are in radar imaging [8,9], DNA microarrays [10,11], surface metrology [12], and biomedical imaging [13,14] including magnetic resonance imaging (MRI) [15–17], computed tomography (CT) imaging [18–20], ultrasound imaging [21–23], and fluorescent imaging [24,25].

As the CS paradigm invokes simultaneous sensing and compression, conventional architectures cannot be used for the implementation of CS encoding. This is because in standard cameras, the level of the light for each spot in the scene, *i.e.*, the pixel value, is captured while the CS technique requires some transformed version of the light level of the entire scene. Therefore, new imaging structures have been proposed to implement CS-based imaging systems.

The first implementations of CS were in the optical domain. Here, the required transformation can be applied to the reflected light coming from the object. The resulting transform coefficients can be sensed by regular sensors and used in the decoder side to recover the pixel values for the image. However, the implementation can be performed in electrical domain as well. The recent advances in CMOS technology have provided for the development of smart imaging systems and the possibility of on-chip signal processing for CMOS-based sensors. Therefore, the CS method can be implemented in the focal-plane with a CMOS sensor in electrical domain. Although the electrical implementation does not lead to less image sensors, the shorter acquisition time can be achieved with a smaller camera size compared to the optical-domain CS cameras.

In this paper, different hardware implementations of the CS-based image acquisition encoding in optical and electrical domains for image and video coding are discussed. In Section 2, the compressive sensing framework including encoding and decoding schemes is discussed. A study of different hardware implementations of CS for image acquisition is presented in Section 3. Section 4 includes the CS system for video coding. Finally, in Section 5, the conclusions are presented.

## 2. Compressive Sensing Framework

### 2.1. Encoding

Natural images consist of highly structured information leading to a strong dependency between the adjacent pixels in the image. This dependency can be mathematically investigated by evaluating the expansion coefficients of the image in a special basis, ψ, e.g., discrete cosine transform (DCT), discrete wavelet transform (DWT), and contourlet transform. Considering an *N*-pixel image as the vector x which is made by column concatenation of the image, then the transformation of the image in basis ψ is:

(1) θ=ψx

where θ =(*θ*_1_, *θ*_2_, …, *θ_N_*) is the vector of expansion coefficients. The image x is called *k*-sparse in ψ domain when only *k* expansion coefficients are significant and the other (*N*-*k*) coefficients are zero or negligibly small. Therefore, although the image is represented by *N* pixel values in grey level domain, it could be represented by *k* values in ψ domain.

Conventional transform coding schemes apply the transform ψ to the image and keep the *k* significant coefficients as the compressed version of the image. The image can be recovered in the decoding process using these coefficients and the inverse transform ψ^−1^. However, all *N* pixels should be acquired to extract the significant coefficients. Therefore, it is not efficient to capture *N* values while only *k* values are enough for the image reconstruction. Compressive sensing addresses the problem of inefficient sampling and then compression.

By sampling all of the pixels separately, their common information is sampled more than once. Although, this extra information can be removed later using image compression algorithms, in compressive sensing, only the necessary information is sampled from the beginning. Therefore, instead of sampling the level of the light from the different points in the scene, the projections of the level of the light for the entire scene are used as the CS measurements. These measurements can be used to recover the image that would be achieved by regular sampling schemes.

CS encoding is performed by acquiring several linear measurements from the image using different projection functions. Considering the image as the vector x, different projections of the image can be shown as follows:

(2) $$y_{1}=\varphi_{1}.\text{x},\;y_{2}=\varphi_{2}.\text{x},\ldots ,\;y_{M}=\varphi_{M}.\text{x},$$

where *y*_1_, *y*_2_, …, *y_M_* are the CS measurement values and *φ*_1_, *φ*_2_, …, *φ_M_* are the projection functions. Each measurement is a weighted-sum of the level of the light on different pixels. By putting all the measurements together, the entire CS encoding algorithm can be expressed by the following linear equation:

(3) $$y_{M\times 1}=\Phi_{M\times N}x_{N\times 1},$$

where *y* is the measurement vector, *M* is the number of measurements and *N* is the number of the pixels in the image. Therefore, the acquisition of *M* measurements instead of *N* pixel values leads to the compression ratio of $\frac{M}{N}$.

As can be seen in Equation (3), the encoding process is a simple matrix multiplication. However, the important part is that the measurement matrix *Φ* should be properly chosen to extract the maximum information from the image. The measurement matrix can be chosen based on the particular properties of the image. However, the structure of the image is unknown, unless CS is used for the compression of an available image, but this is not its main purpose. Also, the structural information of the image might change from one image to another. Therefore, a universal measurement matrix which is applicable for all images is required.

It has been shown that when the number of the measurements (M) is greater than the order of sparsity of the image (k), then the necessary and sufficient condition for image reconstruction is the validity of the restricted isometry property (RIP) for the measurement matrix [5,26,27]. The RIP property is expressed as follows:

(4) $$\left(1-\delta )<\frac{{\left\|\Phi \psi^{-1}v\right\|}_{2}}{{\left\|v\right\|}_{2}}<(1+\delta \right)$$

where *v* could be any vector with the same non-zero coefficients as *θ*, and 0 < *δ* < 1. It can be shown [3] that when $\delta <(\sqrt{2}-1)$, there is an upper bound on the reconstruction error in the image recovery problem. This means that the image can be reconstructed. Also, the measurement matrix should be incoherent with the basis [5]. These properties will be satisfied when the entries of the measurement matrix are independent and identically distributed random variables. Therefore, the CS encoding process can be expressed as the extraction of a set of weighted addition with random weights. By choosing the entries from a set of uniformly distributed binary random variables, the measurement process is the random selection of the pixels in the image and adding the levels of the light for the selected pixels.

### 2.2. Decoding

Reconstruction algorithms use the measurement vector, *y*, the measurement matrix, *Φ*, and the basis, *ψ*, to recover the value of each pixel in the image. The reconstruction methods search for the sparsest vector such that its projection on the measurement matrix domain matches the available measurement values. The 0-norm is the best criterion function to measure the sparsity of a vector. Therefore, the problem can mathematically be expressed as follows:

(5) $$\begin{array}{ccc} {\text{Minimize}}_{x}{\left\|\psi x\right\|}_{0} & \text{subject}\;\text{to} & y=\Phi x. \end{array}$$

The 0-norm minimization is an NP-complete problem. However, it can be shown that the 1-norm minimization, basis pursuit (BP) [2], and the linear programming algorithm can be used for reconstruction if the measurement matrix satisfies the restricted isometry property [26,27]. Therefore, considering the effect of the noise on the measurements values, the reconstruction problem can be shown to be:

(6) $$\begin{array}{ccc} {\text{Minimize}}_{x}{\left\|\psi \text{x}\right\|}_{1} & \text{subject}\;\text{to} & {\left\|\text{y}-\Phi \text{x}\right\|}_{2}<∊ \end{array}$$

where **∊** is an upper bound for the noise level in the measurement process. The problem can also be modified by adding new constraints to improve the quality of reconstruction [28].

Convex programming can be exploited to solve the above optimization problem. Although linear programming leads to promising results in terms of the reconstructed image quality, it is of high computational complexity, for instance in order of $\text{O}\left(M^{2}N^{\frac{3}{2}}\right)$ [29]. Some other fast convex programming methods, for example gradient methods [30], have been presented to improve the computational load of the algorithms. Also, for the optimization problem, some efficient iterative methods based on solving several optimization sub-problems with sparsity regularizer have proposed in [31,32] to improve the convergence time.

There is another family of algorithms based on iterative greedy methods that are computationally more efficient compared to BP methods. However, the cost is a lower image quality for the same number of measurements [33–38]. Greedy methods use iterative, non-optimization approach by successive approximation of the data and its residuals. Orthogonal matching pursuit (OMP) [33], stagewise OMP (StOMP) [34], and regularized OMP (ROMP) [35] are some of the greedy methods with computational complexity in order of O(*KMN*) [36]. Subspace pursuit (SP) [36], compressive sampling matching pursuit (CoSaMP) [37], and adaptive sparsity matching pursuit (ASMP) [38] are some of the other greedy methods with comparable reconstruction quality to the BP methods.

## 3. Hardware Implementation

### 3.1. Single Pixel Cameras

Conventional cameras represent the captured image in spatial domain by using multiple photodetectors as the pixels of the image [39]. Therefore, the resolution of the image increases with the number of photodetectors. However, the first implementations of a CS imaging system used a single photodetector to capture different CS measurements from the scene. Optical devices were used to represent the pixels of the image. Therefore, the resolution of the image was defined in the optical domain.

**Figure 1.**
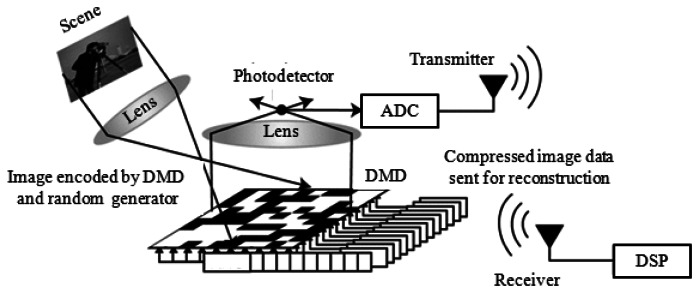
General schematic of single-pixel camera set-up (adapted from [41]) (DMD: digital micro-mirror device; ADC: analog-to-digital converter, DSP: digital signal processor).

The digital micro-mirror device (DMD) and the CS paradigm were used to implement one of the first single-pixel cameras [40,41]. Figure 1 shows a general schematic of the implemented single-pixel imaging systems. Each mirror in the DMD array is tilting towards or away from the photodetector based on its applied random coefficient [42]. The aggregate level of the light, which is integrated by the photodetector, represents one CS measurement. Different measurements are then achieved by different random alignments of the micro-mirrors. A multi-pixel image is recovered using all measurement values and the CS reconstruction methods described in Section 2.2. The idea of single-pixel imaging is also used in compressive confocal microscopes [43,44].

In [45], a single detector terahertz imaging system is presented. A series of random masks have been exploited to make the CS measurements. Each mask represents a random pattern using a combination of transparent and opaque areas corresponding to random coefficients. In [46], structured light and a single detector have been used to measure specific features of the image. Instead of using uniform illumination for imaging, a set of spatially structured illumination patterns have been used to extract a sequence of measurements from the image.

All the single-pixel cameras described above are based on serial measurement extraction. Different measurements are made during consecutive time intervals. Therefore, the object should be stationary during the measurement process. This stationary requirement makes the designs unsuitable for video acquisition when there is movement in the scene from one measurement to the next.

### 3.2. Coded Aperture Cameras

Typical cameras use pinhole apertures and lenses to focus the incoming light from the object onto the sensor array. Therefore, each sensor in the array represents a pixel of the image. However, the optical field from the scene could be modulated using a coded aperture. Therefore, some specific image transformations can be implemented in optical domain [47]. Considering the CS measurements set as a random transformation of the image, then coded aperture can also be used in CS cameras. In [48], a high resolution coded aperture with a low resolution focal plane array aperture has been used for imaging. The aperture has been designed so that different CS measurement values are collected by the different pixels of the low resolution array. Therefore, the value measured by each sensor on the focal plane array is a CS measurement value formed in optical domain by the coded aperture. Contrary to the single-pixel imager, all CS measurement values are extracted in the same time interval.

**Figure 2.**
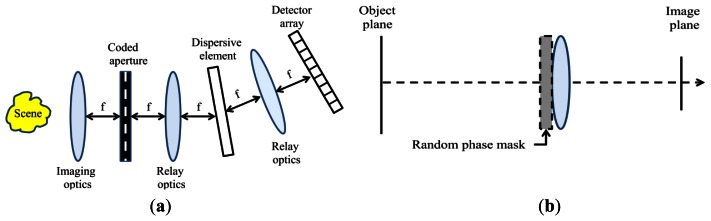
(**a**) Schematic of single dispersive spectral imaging system (adapted from [49]); (**b**) Optical set-up for random-mask image acquisition (adapted from [51]).

The coded aperture approach is also used for spectral imaging. In [49], a single shot spectral imager using the CS framework is presented. To improve the signal-to-noise ratio (SNR) of reconstruction, a multi-shot approach with multiple apertures and focal plane measurement sets is presented in [50]. The CS measurement can also be implemented by using a random mask placed on the lens. Here, the convolution of the image and mask signal is made at a single exposure to create the CS measurement values [51]. Figure 2 shows the spectral imaging structure [49], and the random mask set-up presented in [51].

### 3.3. Random Lens Imaging

An imaging system developed in [52] utilizes random lens made from multi-faceted mirrors for compressive image acquisition. The system is the same as in conventional cameras, but the input-output relationship of the light rays is randomized. The only extra hardware is some small mirror patches stitched around the sensors, thus leading to an ultra-thin design for multi-spectral and high dynamic range imaging [52].

### 3.4. CS-CMOS Cameras

In the hardware implementation of CS encoding discussed in Sections 3.1–3.3, the random transformation required for CS measurements is performed in the optical domain. This leads to an optimum usage of photodetectors to achieve high resolution imaging using low resolution sensor arrays at the cost of a larger camera size due to the optical components. However, the CS method can also be useful as an image compression method because of its straightforward encoding process. Due to the limitation in power and area, compression methods with simple encoding processes are of high interest for on-chip image compression in CMOS imaging systems. Therefore, on-chip implementation of CS algorithms in electrical domain would be useful for portable applications where there are limitations on camera size and power consumption.

**Figure 3.**
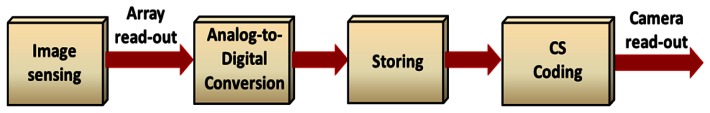
Block diagram for the digital implementation of CS coding.

The electrical domain implementation of CS algorithm could be performed in digital domain. In this case, after the sensing and analog-to-digital (A/D) conversion, the digital values should be stored and then added together with random weights to make the CS measurements. A schematic representation of the digital implementation is shown in Figure 3.

**Figure 4.**
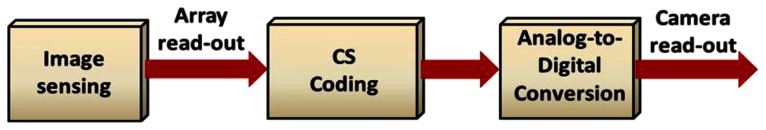
Block diagram for the analog implementation of CS coding.

Although the camera read-out time decreases in this implementation, the array read-out time and amount of the storage are the same as an imager with no compression. Also, the multiple memory accesses for the digital calculation increases the overall read-out time. However, all or some parts of the compression could be performed in analog domain before or as a part of the analog-to-digital conversion. This structure decreases the amount of the on-chip storage area. Also, the analog implementations of the arithmetic circuits are faster and require less area compared to the digital circuits. Figure 4 shows the block diagram representation for the analog implementation.

Several works have been presented on computational imaging and analog signal processing in image sensors [53,54]. These designs are intended to implement the image transformation in analog domain. However, they could be used as a CS encoder by choosing appropriate transform coefficients.

**Figure 5.**
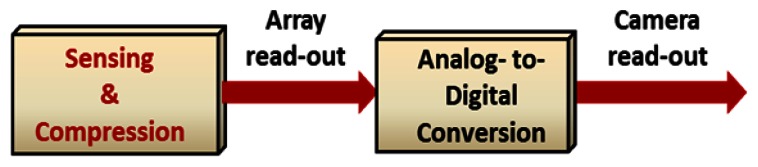
Block diagram of a CS encoding implementation.

The main advantage of the CS encoding, which is the simultaneous sensing and compression, is disregarded for both structures in Figures 3 and 4. To be compatible with the CS concept, the compression should be performed in the array level and before the array read-out. Figure 5 shows the block diagram of a CS imaging system which is well-matched with the CS concept. In this implementation, only the compressed version of the image is read-out, so the entire imaging process is faster and more power efficient.

One of the major difficulties in realizing the CS-CMOS implementation shown in Figure 5 is the method of applying random coefficients to the pixels. The random selection and summation of the pixels can be performed after the light integration [55–57], but this is not based on the block diagram in Figure 5. In [55], a block-based CS encoding for digital pixel sensors (DPS) has been presented. The sensor array has been divided into different sub-blocks and one pixel is randomly selected in each block as the CS measurement for that block. The selected digital outputs are then saved in 8-bit memories to be used in the CS reconstruction process.

Another block-based implementation of CS at the A/D conversion level has been presented in [57]. The random measurement is performed with a CS multiplexer before a ΔΣ-based A/D converter. Row and column block selectors are used to connect different blocks to a CS multiplexer and a pseudo-random generator provides the random coefficients required for the CS multiplexer. Although the measurement process is not performed in parallel with sensing, the different measurements are extracted in parallel. This leads to a single-shot imaging scheme. However, to perform the simultaneous sensing and compression, the random sequence should be applied at the beginning of the integration time to make sure that the corresponding CS measurement is integrated during the light integration. Therefore, the technique of random selection of the pixels is important. Examples of the pixel selection techniques are discussed in the following three subsections 3.4.1 to 3.4.3.

#### 3.4.1. In-Pixel Random Generators

Some designs use in-pixel memories or digital circuits to put all or part of the random generator inside the pixel. However, by putting non-photosensitive elements inside the pixel, the fill factor and sensitivity of the imager decreases [58,59]. Also, the size of the pixel increases, thus leading to lower image spatial resolution.

In [60], in-pixel memories with control logic, a finite state machine (FSM), three linear feedback shift registers (LFSRs) and a single analog processing unit were used to perform in-pixel convolution in real-time. Each pixel contains a three-transistor active pixel sensor (APS) [61,62] with one flip flop as the local memory plus control logic to implement the horizontal and vertical shifting. Using a two-dimensional scrambling technique, the random coefficients are applied in two dimensions over the entire array. Also, the measurement process is performed in two dimensions using the differential current output of each pixel which are connected to the corresponding outputs from other pixels. Finally, the accumulated differential currents are fed to a transimpedance amplifier (TIA) to calculate the final CS measurement. The measurement process is completely performed during the light integration in analog domain, although the scrambling technique needs extra time between the acquisitions of one measurement and the next.

#### 3.4.2. Column-Row Random Selection

Random coefficients can be fed to the pixels from outside the array. However, because of layout restrictions, having individual access to each pixel is impractical for a large array sensor. It is because of the limited number of the metal layers in present-day standard CMOS technologies and the fact that the metal layers cannot pass through the photosensitive areas [63]. Therefore, different sub-regions of the pixels should share common paths to access the coefficients. Random coefficients can be partially fed to the array along the columns so that pixels in each row have the same coefficient. Then, the outputs of the columns are randomly combined outside the array to complete the random measurement.

In [64,65], passive pixel sensors (PPS) [66] were used to design a separable-transform image sensor which is also capable of implementing the CS encoding. Instead of sensing the pixel values for the image, the imager projects the image on a specific basis and produces the projection coefficients. The convolution and image transformation are performed for separate sub-regions of the image with 8 × 8 and 16 × 16 block sizes, respectively. The main capability of the design is to exploit the separability property to perform the image transformation in two steps:

(1) focal plane processing along the columns and during the sensing, and
(2) analog computational units outside the array and before the analog-to-digital converter.

**Figure 6.**
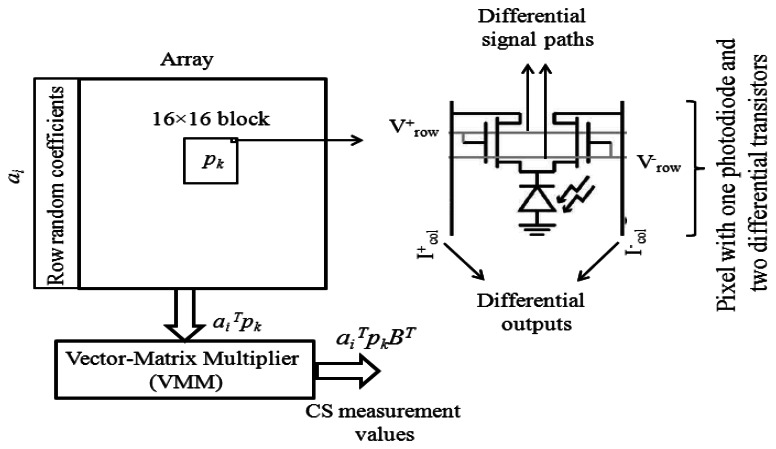
General schematic representation of the separable-transform imager (adapted from [64]).

Figure 6 shows the schematic representation of the imager in [64]. The image transformation is divided into two separate matrix multiplications. The first multiplication is performed by using differential transistors in the pixels and Kirchhoff's current law along the columns. The coefficients for each pixel are provided by the differential signal paths which are the same for the pixels in each row. The results for different columns are fed to an analog vector-matrix multiplier (VMM) to perform the second part of the matrix multiplication. The imager can be exploited for CS imaging by dividing the random multiplication of CS encoding into two separate matrix computations. However, the separability property and analog multiplication is too complicated for the CS implementation, although it is useful in performing other image transformations.

The APS and switched capacitor circuits are used in [67] to implement a separable transform imager. By adding one transistor and one capacitor to the three-transistor APS structure and using a switched capacitor circuit for each column, the random combination of the pixels for each column is achieved. The calculated values from all columns are fed to another switch capacitor circuit to make the final random measurement. The timing of the switching clocks can be adjusted to choose one coefficient from the set {0, +1, −1} for each pixel.

An implementation of CS encoding using random convolution was presented in [68]. The structure is a combination of in-pixel and column-row random selection. Binary random coefficients were provided by in-pixel memories and a LFS) to feed the initial values for the in-pixel memories. Each pixel contains a photodiode and a 1-bit memory which is connected to the memories in adjacent pixels to make an embedded shift register inside the array. The outputs of the pixels in each column are connected together, and Kirchhoff's current law applies along the columns to make a random light integration for each column. A time-domain multiplexer and an analog-to-digital converter are used to digitize the outputs for different columns. The rest of the measurement process is performed by doing several digital summations. To complete the randomness of the convolution process, a pseudo-random triggering LFSR was used after column integration, but before column read-out. Note that the CS measurement process has been performed in two steps, with the second step in digital domain and after the light integration. This is not the simultaneous sensing and compression expected in CS encoding.

#### 3.4.3. Block Random Selection

Column-row random selection provides the possibility of feeding random sequences from outside the array by using the same signal paths for pixels in each column. However, the metal paths could be shared among different blocks of the images, instead of the columns. As the size of the block is small compared to the entire array, then the individual access to all pixels in the block from outside the array is feasible. Also, each pixel in the block could share the metal path with its corresponding pixels in other blocks. Therefore, each random coefficient is connected to all the corresponding pixels in different blocks. It is analogous to the column-row structure when all pixels in one column share the same random coefficient. Therefore, the array can be divided into separate blocks and the CS encoding can be implemented for different blocks separately. Individual access to all pixels in each block can be possible at the time of measurement time of the block.

**Figure 7.**
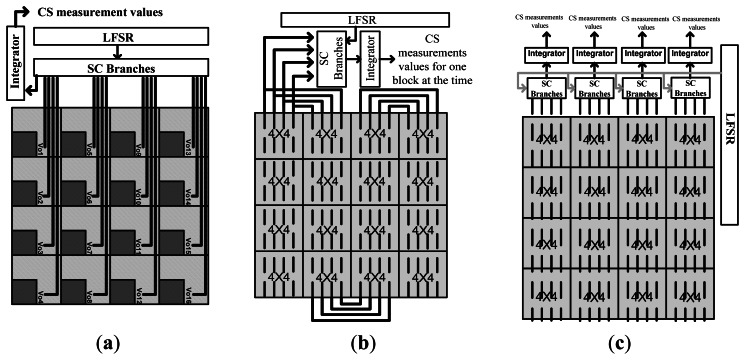
(**a**) Connections of each block toward the linear feedback shift register (LFSR); (**b**) Block connections for block-by-block read-out; (**c**) Block connections for column-of-blocks connections (adapted from [63]).

In [63], a new block-based implementation of CS using a three-transistor APS structure, an off-array LFSR, and switched capacitor (SC) circuits, was designed. The SC branches and LFSR are designed outside the array and there is no extra in-pixel element for compression. The random coefficients produced by the LFSR are fed to the SC branches outside the array. However, to provide the individual connection of pixels and SC branches, the imager is designed in different blocks which share the same signal paths toward the SC branches. Figure 7(a) shows a block diagram of the connection between the pixels, SC branches and LFSR.

The connections between different 4 × 4 blocks in the array for block-by-block read-out are shown in Figure 7(b). In this structure, there is one set of SC branches for the entire array. Therefore, the CS measurement is performed for one block at a time. To improve the read-out time of the array, the measurement process can be performed in parallel for different columns of blocks, as shown in Figure 7(c). There is one integrator and one set of SC branches for each column of the blocks. Therefore, the measurement process is performed in parallel at the same time, for different columns of blocks.

Table 1 shows a summary of different CS-CMOS imager architectures discussed in this paper. The implementation can be in analog or digital domain. In the digital implementation, the random selection and measurement extraction are straightforward as they use the digital data outside the array. However, the analog implementation leads to a faster and more efficient encoding process. As can be seen in the table, different works use different methods of random selections. In-pixel random selection leads to the use of extra digital components inside the pixel which reduce the fill factor of the design. However, column-row and block random selection methods use the external random coefficients to avoid the use of internal digital circuits. Also, the block method is more efficient in terms of in-pixel and overall hardware usage.

## 4. CS Video Capture

**Figure 8.**
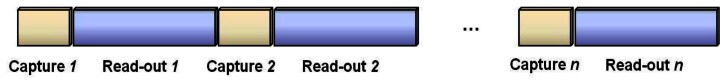
Timing diagram for frame-by-frame video coding using consecutive image coding steps.

The structures mentioned in the previous section can be used for video acquisition by encoding each frame of the image separately. In this case, the measurement values for each frame should be captured and sent off the camera before starting the measurement process for the next frame. The entire video acquisition scheme for *n* consecutive frames of the video is shown in Figure 8.

The spatial dependency of different pixels in the image is exploited as the sparsity objective function in the CS image decoding process. However, there are temporal correlations between the consecutive frames of a video which can be used to reduce the reconstruction time of a CS-encoded video.

In [69], the temporal information of the video was exploited to improve the overall reconstruction time. In one implementation, the decoded image for each reconstructed frame was used as the initial value for the reconstruction problem of the next frame. In another implementation, the CS decoding problem was solved for multiple frames at the same time with reasonable computation time by using the similarities between the subsequent frames.

While only the sparsity of the image derivative in temporal domain has been used in [69], the motion function of the video from one frame to another can be used to improve the reconstruction. In [70], considering the relationship between motion and CS coding, a method to estimate the motion information of the image was presented. Also, the motion parameters were included in the reconstruction problem to improve the quality of decoded frames.

**Figure 9.**
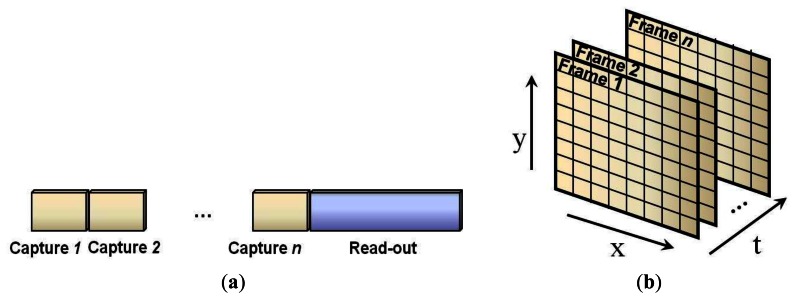
(**a**) Multiple-capture and one read-out timing diagram; (**b**) Cube of data for *n* frames.

Considering the timing diagram in Figure 8, the video frames cannot be captured during the read-out intervals. The fact that the read-out time increases with increasing imager size restricts the frame rate of the CS video camera. However, the measurement process can be performed in temporal domain as well as in spatial domain to improve the capture time of a constant number of frames. Therefore, the measurements can be captured for a three-dimensional cube of data instead of separate measurements for several two-dimensional data sets, *i.e.*, image frames, as shown in Figure 9. Using the measurement process in Figure 9(a), the read-out time of the camera is reduced by the ratio of 1/*n*.

A new CS measurement method for video compression based on the read-out scenario shown in Figure 9 was presented in [71]. In this method, multiple cameras capture the CS measurements from the same scene. Each camera then produces a frame of the measurement values instead of *n* frames of pixel values. Different measurement frames captured from different cameras are exploited to reconstruct the video for *n* subsequent frames.

The measurement process is performed completely in time domain. One binary random vector with *n* entries is assigned to each pixel of the image in each camera. If the *i^th^* bit of the vector is “1”, the level of the light for that pixel in the *i^th^* frame is integrated and added to the measurement value for that pixel. The level of the light during each frame does not affect the measurement value when its corresponding bit in the random vector is “0”.

The simulation results for the image reconstruction are promising and lead to a high frame rate video recording system [71]. However, regular sensor structures cannot be used for the hardware implementation of this design. New pixel structure should be considered to perform the CS measurement process in time domain. Also, since each pixel needs a separate random sequence, new array architecture is required to apply the random bits to the pixels.

## 5. Conclusions

In this paper, we reviewed compressive sensing (CS) image acquisition with encoding and decoding techniques, and discussed several hardware implementations. Different implementations in optical domain by using digital micro-mirror device (DMD) arrays, coded aperture or random lens systems in early implementation of CS encoding were reviewed. However, the complexity of the optical components and the size of the camera make the optical implementation of the CS encoding unsuitable for portable applications. Also, because of the significant advances in *complementary metal-oxide-semiconductor* (CMOS) technology, the implementation of CS on focal plane of the CMOS sensors is very feasible. Therefore, we reviewed recent implementations of CS encoding in electrical domain for CMOS sensor structures. In addition, recent works on CS video coding were discussed.

## Figures and Tables

**Table 1.** Summary of various CS-CMOS imagers available in the literature.

**Ref.**	**Pixel Type**	**Tech. (cmos)**	**Array Size**	**In-pixel Components**	**Technique**
[55]	DPS	--	--	1 transistor, 2 inverters, 1 AND, 1 comparator	Digital domain, Random selection after integration
[57]	APS	0.15 μm	256 × 256	4 transistors	Analog-to-digital conversion level, Random selection after integration
[58]	APS	0.18 μm	256 × 256	3 transistors, 3 NAND, 1 D-flip flop	Analog domain, In-pixel random selection, Differential current & Trans-impedance amplifier
[63]	APS	0.13 μm	16 × 16	3 transistors	Analog domain, Block random selection, Switched capacitor (SC) circuits
[64]	PPS	0.35 μm	256 × 256	2 transistors	Analog domain, Column-row random selection Differential current & Vector matrix multiplier
[67]	APS	0.5 μm	128 × 128	4 transistors, 1 capacitor	Analog domain, Column-row random selection SC circuits
[68]	PPS	--	--	1 transistor, 1 flip flop	Analog & digital domain, In-pixel & column-row random selection
